# Combined Effects of Muricid Extract and 5-Fluorouracil on Intestinal Toxicity in Rats

**DOI:** 10.1155/2015/170858

**Published:** 2015-05-13

**Authors:** Roger Yazbeck, Ruth Lindsay, Catherine A. Abbott, Kirsten Benkendorff, Gordon S. Howarth

**Affiliations:** ^1^Sansom Institute, University of South Australia, Adelaide, SA 5000, Australia; ^2^Basil Hetzel Institute, Queen Elizabeth Hospital, Woodville, SA 5011, Australia; ^3^Gastroenterology Department, Women's and Children's Hospital, Adelaide, SA 5000, Australia; ^4^School of Animal and Veterinary Sciences, The University of Adelaide, Roseworthy Campus, Adelaide, SA 5371, Australia; ^5^School of Biological Sciences, Flinders University, Adelaide, SA 5000, Australia; ^6^Flinders Centre for Innovation in Cancer, Flinders University, Adelaide, SA 5000, Australia; ^7^Marine Ecology Research Center, School of Environment, Science and Engineering, Southern Cross University, Lismore, NSW 2480, Australia

## Abstract

Chemotherapy drugs, such as 5-fluorouracil (5FU), are the standard approach for cancer and are associated with several peripheral toxicities. We previously demonstrated that Muricidae marine molluscs exhibit chemopreventive properties. This study investigated the combined effect of muricid extract derived from* Dicathais orbita*, with 5FU, on intestinal toxicity in rats. Groups of rats were orally gavaged water, muricid extract, or sunflower oil, with or without 5FU (150 mg/kg). Metabolic data was collected daily and small intestinal brush border enzyme activity was measured by sucrose breath test (SBT). Blood was collected by cardiac puncture for whole blood analysis. Intestinal biopsies were taken for histopathology. Neutrophil activity was measured by myeloperoxidase activity. No additional toxicity effects were observed in rats receiving the combination of 5FU and muricid extract compared to 5FU alone, as indicated by SBT, histopathology, and myeloperoxidase activity. Intestinal integrity was protected from 5FU-induced damage in the sunflower oil vehicle group, compared to controls, as measured by SBT, villus height, and crypt depth. We concluded that combination of muricid extract and 5FU did not confer any additional intestinal toxicity, further supporting its potential as a chemopreventive food product. In this model system, sunflower oil partially protected against 5FU-induced intestinal toxicity.

## 1. Introduction

Colorectal cancer (CRC) is the second most common malignancy in the world and has the second highest mortality rate of all cancers [1]. Pathogenesis is initiated by the formation of aberrant crypt foci, mucosal cell clusters, polyps, adenomas, and adenocarcinoma [1]. Treatment with 5-fluorouracil (5FU) and leucovorin (folinic acid) is the mainstay of therapy for patients with stage III and selected stage II CRC [2]. However, cytotoxic drugs, such as 5FU, are commonly associated with significant peripheral toxicities arising from off-target specificity, targeting all rapidly dividing cells, particularly the gastrointestinal epithelia.

Clinically referred to as mucositis, it affects 40% of patients after standard doses of chemotherapy and up to 100% of patients undergoing high dose chemotherapy for hematopoietic stem cell transplantation, or radiation for head and neck cancers [3]. Symptoms of mucositis include, but are not limited to, dysphagia, ulceration, bleeding, abdominal pain, and diarrhoea [3]. Furthermore, the damaged intestinal barrier may allow for the translocation of pathogens, leading to life-threatening complications with septicemia [4]. These dose-limiting and potentially life-threatening symptoms impact patient quality of life but also lead to a significant economic burden on health care systems [3]. Novel therapeutic agents possessing chemopreventive properties, with limited or no gastrointestinal toxicities, are urgently required.

Recently, natural food products have emerged as potential chemopreventive agents for a range of different cancer types [5–8]. We have recently identified Muricidae marine molluscs as a source of natural brominated indole derivatives with potential chemopreventive properties [9]. At relatively high concentration, crude muricid extracts derived from* Dicathais orbita*, containing a mixture of 6-bromoisatin and tyrindoleninone (6-bromo-2-methylthio-3H-indol-3-one), were found to inhibit the growth of a range of solid tumour and lymphoma cell lines [10, 11]. In particular, these molluscs extracts reduced the viability of the HT29 and Caco2 colon carcinoma cell lines by over 70% and 80%, respectively* in vitro *[10]. Semipurified extracts containing tyrindoleninone were cytotoxic towards Caco2 cells (IC_50_ = 98 *μ*M), and HT29 (IC_50_ = 390 *μ*M), whereas 6-bromoisatin induced cell death by apoptosis in both cell lines at approximately 100 *μ*M [9]. Furthermore, in mice with azoxymethane induced CRC, prophylactic oral administration of high dose (1 mg/g) muricid extract, containing these brominated indoles, displayed significantly higher rates of apoptosis in the distal colon relative to control mice [12]. The purple dye secretion from muricid molluscs also contains the minor pigment 6,6′ dibromoindirubin [13], which has been identified as a potent protein kinase inhibitor [14–16]. These indirubin derivatives have been shown to induce apoptosis in human cancer cells [17–19]. Furthermore, indirubin has been shown to have inhibitory effects on the production of interferon-*γ* and to suppress inflammatory reactions in delayed type hypersensitivity in mice [20]. Thus, Muricidae derived products represent potential chemotherapeutic or chemopreventive reagents.

In many cultures, marine molluscs, including Muricidae whelks, are regarded as a healthy food, featuring in a range of traditional natural remedies [9]. Muricidae molluscs form a traditional component of some African, European, Mediterranean, and Asian diets [21–23], indicating their tolerability for human consumption. Nevertheless, the potential for toxicity associated with a concentrated bioactive extract from these molluscs requires further consideration.

Our preliminary studies of a Muricidae extract indicated the potential for mild, idiosyncratic effects on the liver and gastrointestinal tract in a small proportion of mice [24]. Furthermore, it is likely that, in clinical use, the extracts would be used as an adjunct to traditional chemotherapy agents, aimed at lowering cytotoxic doses and in turn harmful side-effects, primarily mucositis. The current study aimed to define the potential toxicity effects of muricid extract in rats, when administered alone, or in combination with 5FU.

## 2. Methods

### 2.1. Preparation and Analysis of Extract

Frozen* Dicathais orbita* were cracked in a vice, to allow removal of the shell and access to the hypobranchial glands, which were excised for extraction according to Westley et al. [12]. Hypobranchial glands were extracted by soaking in AR grade chloroform and methanol (1 : 1, v/v, Selby-Biolab, Pronalys, Melbourne, VIC, Australia) under agitation at 4°C for 2 h in a dark room, followed by overnight soaking in fresh solvent and a final 2 hr extraction. Milli Q water was used to induce the separation of the chloroform layer from the more polar compounds and the chloroform extract was dried by rotary evaporation at 40°C in a quickfit flask covered with alfoil to exclude light. The extract was then transferred to preweighed amber vials and dried under a stream of high purity N_2_ gas and stored at −80°C until use. Extract was dissolved in sunflower oil to facilitate oral administration.

The extract was analyzed using liquid chromatography coupled with mass spectrometry (LC/MS) using the procedures previously established in our laboratory [25]. The extract was dissolved in acetonitrile and analyzed by HPLC (Waters Alliance) on a Hydro-RP C18 column, coupled to an electrospray ionization mass spectrometer (ESI MS, Micromass, Quatro micro), with parallel UV/Vis diode-array detection at 300 and 600 nm. Mass Lynx 4.0 software was used to analyze the data. The relative percent composition of brominated indoles was calculated from the integrated area under the curve for the intensity (absorbance units) of each peak at 300 nm.

### 2.2. Animal Studies

Female dark agouti rats, sourced from the University of Adelaide (*n* = 72), were housed in individual metabolism cages (Tecniplast, PA, USA), in a temperature-controlled room with a 12-hour light/dark cycle. Animals were allowed* ad libitum* access to water and food (18% weight maintenance casein diet). 1 mL of water or extract dissolved in sunflower oil was administered daily via orogastric gavage between −72 and 72 hours of the experimental period. At 0 hours, all rats were administered a single dose of either saline or 5-fluorouracil (150 mg/kg; Mayne Pharma Pty Ltd, VIC, Australia) via intraperitoneal injection. Rats were randomly allocated to one of eight groups (*n* = 8): Saline injection groups were administered either water, sunflower oil, 0.1 mg/g crude extract (low dose (LD)), or 0.5 mg/g crude extract (high dose (HD)), and 5FU injection groups were administered either water, sunflower oil, 0.1 mg/g crude extract, or 0.5 mg/g crude extract. The 5FU dark agouti rat model of mucositis has been well established [26] and was selected for its resemblance to the physiological manifestation of chemotherapy induced toxicity in humans. This study was carried out in strict accordance with the recommendations of the Australian Code of Practice for the Care and Use of Animals for Scientific Purposes. The protocol was approved by the animal ethics committee of the Children, Youth and Women's Health Service (approval number AE700/3/2010).

Rats were monitored daily and metabolic indices including food and water intake, urine and fecal output, and bodyweight were recorded. Furthermore, stool consistency was monitored daily and scored according to a diarrhoea index, where 0 = well-formed stools to 3 = watery stools. Rats were killed by CO_2_ overdose and cervical dislocation 72 hours after saline or 5FU injection.

### 2.3. ^13^C-Sucrose Breath Test

The ^13^C-sucrose breath test (SBT) was performed to assess small intestinal disaccharidase activity* in vivo *[27]. The SBT was performed prior to the commencement of the trial (−72 hours before 5FU/saline), prior to saline or 5FU injection (0 hours), and prior to sacrifice (72 hours after 5FU/saline injection). Animals were fasted overnight, before being gavaged with 1 mL of 0.25 g/mL sucrose solution, labeled with ^13^C. They were then placed in sealed perspex containers for 2 minutes, before a 20 mL sample of breath was collected into an evacuated tube. Breath samples were collected at 0, 15, 30, 45, 60, 75, 90, 105, and 120 minutes after administration of the sucrose solution. The samples were analysed for ^13^C by an isotope ratio mass spectrometer, as described by Howarth et al. [27], and all data was expressed as a % cumulative dose at 90 minutes (%CD90).

### 2.4. Tissue Collection

Rats were anaesthetized by CO_2_ after which blood was collected by cardiac puncture and stored in heparinized tubes for later analysis. Death was confirmed by cervical dislocation and the gastrointestinal tract of each animal was removed, measured, emptied of contents, and weighed. Segments (2 cm) were removed from the jejunum and ileum and placed in 10% buffered formalin for histological analyses. In addition, segments (4 cm) directly adjacent to the corresponding histological samples were collected and snap frozen in liquid nitrogen for biochemical analysis.

### 2.5. Blood Analysis

Immediately after kill, blood samples stored on ice were subjected to whole blood analysis via the Blood Cell Diagnostic System CELL-DYN 3700 (Abbott Diagnostics). Total white and red blood cells (10^9^/L and 10^12^/L, resp.), as well as differential white blood cell (WBC) count (%), white and red cell counts, haemoglobin levels (g/L), hematocrit (%), mean cell volume (fL), mean cell haemoglobin (pg), mean corpuscular haemoglobin concentration (g/L), red blood cell (RBC) distribution width (% cell volume), platelet numbers (10^9^/L), and mean platelet volume (fL) were quantified by this technique.

### 2.6. Myeloperoxidase Assay

All tissue samples were stored at −80°C, until required for biochemical analyses when samples were homogenized in 10 mM phosphate buffer (pH 6). Myeloperoxidase (MPO) is present in neutrophils and can be used as an indirect marker of small intestinal inflammation. MPO activity was measured using previously described methods [28]. Briefly, samples were centrifuged at 13000 g for 10 minutes, after which the supernatant was discarded, and the tissue homogenate was resuspended in 0.5% hexadecyltrimethyl ammonium bromide. After vortexing for 2 minutes, samples were recentrifuged, at 5000 g for 2 minutes. The supernatants were then dispensed into 96-well plates. After the addition of o-dianisidine, absorbance was read at 450 nm.

### 2.7. Histology

Intestinal samples were transferred from 10% buffered formalin into 70% ethanol 24 hours after collection. Specimens were then routinely processed and embedded in paraffin wax and 4 *μ*m sections prepared and stained with hemotoxylin and eosin. Small intestinal crypt depth and villus height were measured in the jejunum and ileum (40 villi and 40 crypts per section) [29]. Blinded analyses of all samples were undertaken using a light microscope (Olympus BH-2, Tokyo, Japan) and digital camera (Sony, Tokyo, Japan) and Image Pro-Plus Software Package Version 4.5.1.2.7 (Media Cybernetics, Silver Spring, MD).

### 2.8. Statistical Analysis

Daily metabolic data, diarrhoea severity, and SBT data were analysed using a repeated measures ANOVA and pairwise comparisons made by a Bonferroni* post hoc* test with a significance level of 0.05. Histological data, MPO, and blood data were analysed by two-factor (factor 1 = injection type, factor 2 = gavage treatment) ANOVA with a Bonferroni adjustment and a significance level of 0.05. Normal data distribution was determined by Q-Q plots of data sets, and data transformations were applied as deemed appropriate. All data were expressed as mean ± standard error of the mean (SEM). Statistical analysis was conducted using SPSS 15.0.1 for Windows (IBM SPSS).

## 3. Results

### 3.1. Extract Composition

**Figure 1:**
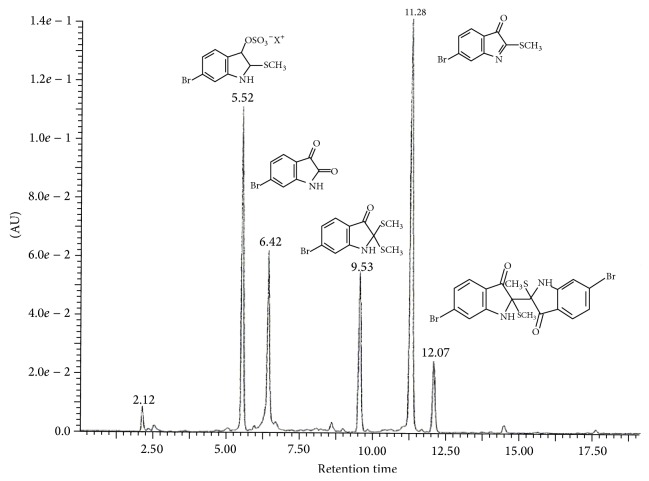
Liquid chromatograph of the extract from the hypobranchial gland of* Dicathais orbita*. The absorbance units (AU) are from the diode array detector at 300 nm. The major peaks have been identified by retention time (r.t.) and characteristic mass isotopic clusters for Br^79^, Br^81^; r.t. 5.52 min = tyrindoxyl sulphate,* m/z* = 336, 338; r.t. 6.42 min = 6-bromoisatin,* m/z* 224, 226; r.t. 9.53 min = tyrindolinone,* m/z *= 302, 304; r.t. 11.28 min = tyrindoleninone,* m/z *= 255, 257; and r.t. 12.07 min = tyriverdin* m/z *511, 513, 517.

Consistent with previous studies [11, 12, 25, 30], the extract from the hypobranchial glands of* Dicathais orbita *was composed of a mixture of brominated indole derivatives. LC/MS analysis revealed that the dominant compound was tyrindoleninone (36.5%, retention time 11.28 min; Figure 1) with a molecular mass of 257, 257 for Br^79^ and Br^81^. Another dominant peak at 5.52 min corresponds to tyrindoxyl sulphate (28.9%, Figure 1), with major ions in ESI-MS at* m/z *336, 338. The peak at 6.42 min with major ions in ESI-MS at* m/z *224, 226 was attributed to the molecular mass of the oxidative product 6-bromoisatin (16.5%, Figure 1), whereas the mass spectrum of the peak at 9.40 min with major ions in ESI-MS at* m/z *302, 304 was indicative of the methane thiol adduct tyrindolinone (11.8%, Figure 1). The smaller peak at 12.07 min can be attributed to the dimer tyriverdin (4.1%, Figure 1), with* m/z *511, 513, 515 (for Br^79^ Br^79^, Br^79^ Br^81^, and Br^81^ Br^81^) and major fragment ions at* m/z *417, 419, 421 formed by the elimination of dimethyl disulphide. A minor peak detected at 14.4 min (0.5%, Figure 1) is consistent with the Tyrian purple pigment 6,6-dibromoindigo and/or 6,6, dibromoindigotin (*m/z/*417, 419, 421) [25]. The minor peaks at 2.12 min are consistent with the choline ester murexin (1.5%* m/z* 224, fragment ion at 165) [9].

### 3.2. Metabolic Parameters

**Figure 2:**
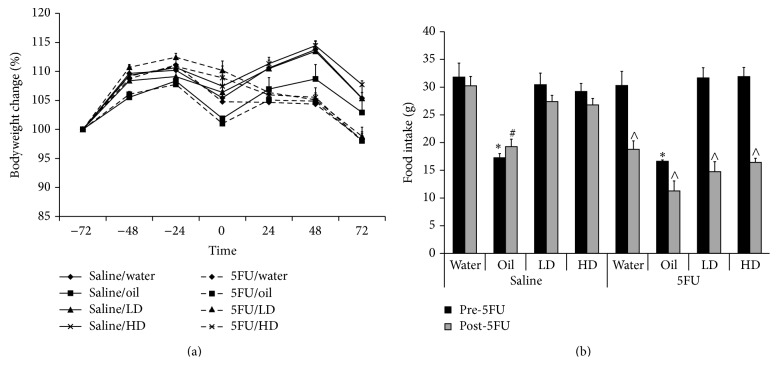
Total food intake in rats. (a) All rats receiving 5FU failed to gain weight after 5FU injection at 0 hr compared to their respective non-5FU controls. (b) Total food intake was decreased following 5FU injection. Furthermore, total food intake was significantly lower (*p* < 0.05) before 5FU in the oil/saline and oil/5FU compared to their respective controls; ^*^oil/saline and oil/5FU compared to water/saline and water/5FU, respectively, ^^^oil/saline compared to water/saline, LD/saline, and HD/saline, and ^^^before 5FU injection compared to after 5FU injection.

Following 5FU injection, bodyweight was significantly lower in all 5FU groups compared to non-5FU controls (Figure 2(a)), consistent with the 5FU model of intestinal mucositis. Food intake (measured as total food intake before and after 5FU or saline injection) was significantly lower in the saline/oil control groups before and after 5FU injection compared to the saline/water control and snail extract treatment groups (Figure 2(b)). Similarly the 5FU/oil group had significantly lower food intake than the 5FU/water control, before 5FU injection; however, there was no difference in food intake between saline/oil and all other groups after 5FU (Figure 2(b)).

There was a moderate increase in diarrhoea severity 72 hours after 5FU injection in all oil treatment groups (1.25), but not in the 5FU/water group (0.08). Furthermore, muricid extract alone did not induce diarrhoea in healthy animals, nor did it increase the severity of diarrhoea in animals injected with 5FU.

### 3.3. Intestinal Health and Integrity

**Figure 3:**
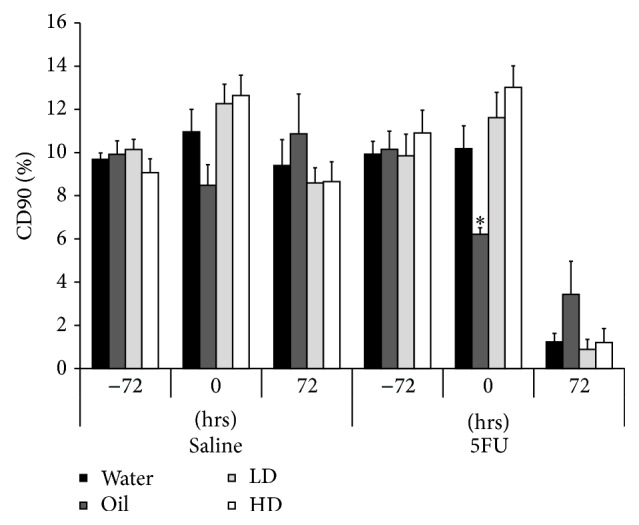
Brush border sucrase activity, measured longitudinally by sucrose breath test. Sucrase activity was decreased in all groups 72 hours after 5FU injection, indicating reduced intestinal function. Sucrase activity was significantly lower in the oil only group compared to all other 5FU groups at the 0-hour time-point. ∗ indicates *p* < 0.05 for oil/5FU compared to all other 5FU groups at 0 hours.

The SBT has previously been validated in rodent models as a surrogate marker of brush-border sucrase activity, and consequently, small intestinal integrity and function [27]. Using the SBT, sucrase activity was measured* in vivo *as a longitudinal marker of intestinal function and villous integrity. At the 0 hour time-point, %CD90 was significantly lower in the sunflower oil gavage group by 23% and 40% in the saline and 5FU cohorts respectively, when compared to water gavage controls (Figure 3).

**Figure 4:**
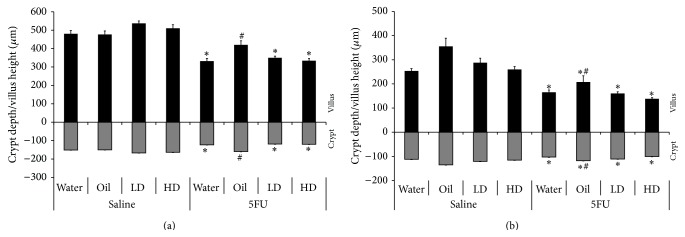
Small intestinal histology data. (a) Jejunal and (b) ileal villus height (above the *x*-axis) and crypt depth (below the *x*-axis) in rats after 5FU injection compared to their respective saline control. ∗ indicates *p* < 0.05 for 5FU groups compared to respective counterpart in saline group. # indicates *p* < 0.05 for oil/5FU compared to all other 5FU groups.

Histologically, the 5FU rat model of intestinal mucositis is characterised by villus and crypt atrophy, manifesting predominantly in the jejunum at 72 hours after 5FU. Accordingly, jejunal villus height was decreased after 5FU injection (Figure 4(a)) by 31%, 35%, and 35% for the water, LD, and HD groups, respectively, compared to their corresponding non-5FU controls (*p* < 0.05). Jejunal crypt depth was also decreased after 5FU by 19%, 29%, and 27% in water, LD and HD groups, respectively, compared to non-5FU controls (*p* < 0.05). Villus height and crypt depth was significantly higher in the oil group after 5FU compared to water, LD, and HD groups. Ileal villus height and crypt depth was similarly decreased in all groups 72 hours after 5FU compared to non-5FU controls (Figure 4(b), *p* < 0.05); however, villus height and crypt depth was significantly higher in the oil group compared to all other post-5FU groups.

### 3.4. Immune Parameters

**Figure 5:**
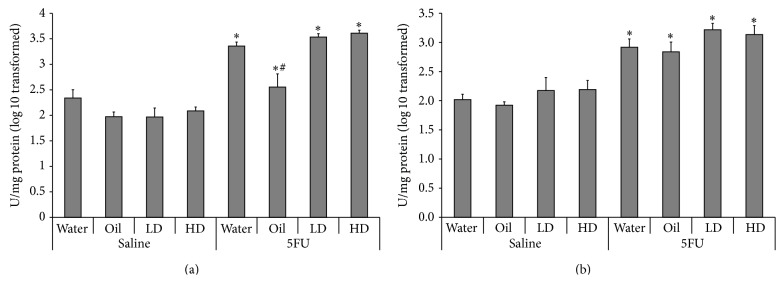
Small intestinal MPO activity. (a) Jejunal and (b) ileal sections 72 hours after 5FU injection. ∗ indicates *p* < 0.05 for 5FU groups compared to respective saline controls. # indicates *p* < 0.05 for oil/5FU compared to all other 5FU groups.

MPO is secreted from activated neutrophils and small intestinal MPO activity is increased during 5FU-induced mucositis in rats [29]. In the current study, jejunal MPO significantly increased in all 5FU groups compared to their respective saline controls (Figure 5(a)). Jejunal MPO activity was significantly lower by 34%, 38%, and 41% in the sunflower oil only group, 72 hours after 5FU compared to water/5FU, LD/5FU, and HD/5FU, respectively (*p* < 0.05).

Ileal MPO was significantly increased in all groups 72 hours after 5FU compared to their respective saline controls (Figure 5(b), *p* < 0.05). There was no difference in MPO activity observed between any of the oil or snail extract treatment groups, within 5FU or saline injected groups (*p* > 0.05).

**Figure 6:**
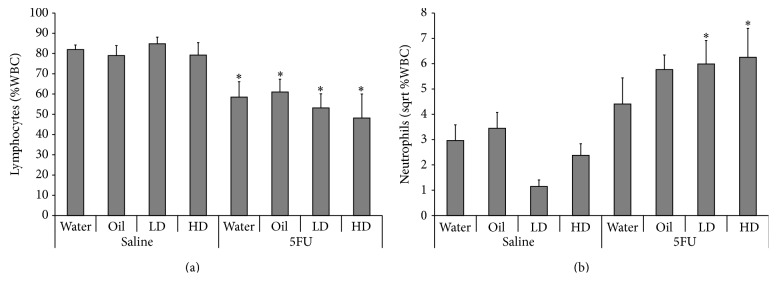
Blood analysis data. Circulating blood (a) lymphocytes and (b) neutrophils expressed as a percentage of total white blood cells in rats, 72 hours after 5FU injection relative to saline controls. ∗ indicates *p* < 0.05 for 5FU groups compared to respective control in saline group.

Whole blood analysis was undertaken to define circulating cell types in blood in response to treatment with the extract. Lymphocyte numbers were lower by 29%, 23%, 37%, and 39% in the water, sunflower oil, LD, and HD post-5FU groups, respectively, compared to their saline-only controls; however, no extract specific response in circulating lymphocytes was observed in 5FU groups (Figure 6(a)). The percent of lymphocytes in the blood was significantly reduced in response to 5FU injection relative to saline controls but there was no significant effect of oral gavage treatment.

Conversely, the percent of neutrophils in the blood tended to increase with 5FU injection relative to saline injected controls (Figure 6(b)) but there was also a significant interaction between oral gavage treatment and 5FU injection. Circulating neutrophils were significantly increased in both the LD/5FU (425%) and HD/5FU (162%) groups compared to their respective saline controls (*p* < 0.05). Circulating neutrophils were also increased in the oil/5FU group relative to saline injected oil gavaged controls (67%); however, this did not reach statistical significance (*p* = 0.058).

## 4. Discussion

We have previously reported the chemopreventive properties of muricid extract, originating from* Dicathais orbita *[12]. In this study, no intestinal or other peripheral toxicities were observed when muricid extract was administered by daily oil gavage to healthy rats for seven days, in the presence or absence of 5FU. In clinical application, it is possible that this “nutraceutical” extract could be used in combination with traditional chemotherapy agents, as an adjunct dietary supplement, rather than a stand-alone therapy. Existing chemotherapy regimens, such as 5FU, can be associated with life-threatening toxicities, particularly gastrointestinal mucositis [2]. Therefore, identifying novel compounds that can target tumour cells with no added toxicities is a priority. The current study found that the combination of the muricid extract and 5FU in rats resulted in no additional intestinal toxicity effects.

Body weight and diarrhoea are commonly used clinical indicators of the extent of intestinal toxicity associated with cancer therapy regimens. In the current study, muricid extract alone or in combination with 5FU did not exacerbate either of these parameters. Furthermore, crypt depth, villus height, brush border enzyme activity, and intestinal neutrophil activity were not modified in rats treated with muricid extract alone or in combination with 5FU. We have previously demonstrated a capacity for muricid extract to increase the crypt cell apoptotic index in the distal colon of mice with azoxymethane induced colorectal cancer [12]. Consistent with the current study, no intestinal toxicity effects were reported, even at the highest dose, suggesting crude muricid extract could be a safe, nutraceutical option during treatment regimens for colorectal cancer. However, Westley et al. [24] reported some mild, idiosyncratic effects on the liver and gastrointestinal tract after 14 days of oral gavage of muricid extract, implying further refinement of the composition and method of administration is still required.

No additional peripheral toxicities were observed in rats receiving snail extract, suggesting that this combination of therapies could be used, in an acute setting, with no detrimental effects. Circulating lymphocytes were decreased in all groups 72 hours after 5FU injection. In saline injected rats, there was a small decrease in circulating neutrophils in both groups receiving muricid extract compared to water and oil only controls. Although not statistically different, the decrease in circulating neutrophils suggested that the muricid extract may have some neutropenic effects. However, in the 5FU injected rats, muricid extract appears to increase the number of circulating neutrophils relative to water and oil controls. The synergistic effect of the extract treated groups injected with 5FU was statistically significant, relative to saline injection, suggesting that the extract has an immunomodulatory effect. A dose dependant increase in the white blood cells was also observed in mice treated with 6-bromoisatin for two weeks and then injected with the carcinogen azoxymethane [31]. Further investigation is required to define whether 6-bromoisatin in muricid extract has a direct or indirect effect in lowering and activating blood neutrophils in the presence and absence of other chemotherapeutic agents.

Lipid extracts from the related Muricidae gastropod* Rapana venosa* have been previously demonstrated to significantly improve the healing of induced skin burns in Wistar rats [32]. Evidence for anti-inflammatory activity was supported by normal blood cell counts in experimental rats treated with* R. venosa *extracts, compared to increasing quantities of leucocytes, lymphocytes, eosinophils, and monocytes in the control rats [32]. Although the bioactive brominated indoles in Muricidae extracts have not been tested for anti-inflammatory activity, the related metabolite, indirubin, can suppress the inflammatory response in mice with delayed type hypersensitivity [20]. Brominated derivatives of indirubin also exhibit inflammatory activity in RAW 264.7 cells [33] and in rat brain microglia [34] and inhibit LPS-stimulated production of tumor necrosis factor, NF-*κ*B, interleukin-1, prostaglandin E2 (PEG2), and intracellular reactive oxygen species [33]. Trace amounts of Tyrian purple pigment were detected in the extract and 6,6′-dibromoindirubin can be generated from tyrindoxyl sulphate and 6-bromoisatin in the presence of oxygen and sunlight. Therefore this compound may have contributed to some of the observed immune-modulatory activity. Furthermore, isatin has been found to inhibit NO production, COX-2, TNF, and PGE2 in mouse macrophages [35]. Therefore, further investigation of the immune-modulatory properties of Muricidae extracts and bioactive compounds in isolation, as well as any synergistic effects in combination, appears to be warranted.

Partial protection from 5FU-induced intestinal mucositis was observed in sunflower oil treated rats. This was reflected by improved villus height and crypt depth measurements in the jejunum and ileum, as well as lower jejunal MPO activity compared to all other 5FU groups. Several studies have previously demonstrated the protective capacity of animal and plant derived oils using animal models of intestinal damage. For example, emu oil is derived from the subcutaneous and retroperitoneal fat of emus and has been used topically for pain relief and to accelerate wound healing [29]. Recently, a daily oral gavage of emu oil has shown some efficacy in rat models of intestinal mucositis and colitis, increasing villus height and crypt depth, and reducing neutrophil activity [29, 36]. Similarly, daily oral gavage of Lyprinol, an extract of the New Zealand green lipped mussel, has demonstrated anti-inflammatory activity in a mouse model of colitis [37]. Villus height and crypt depth was also increased in rats consuming olive oil or maize oil* ad libitum* in their diets compared to respective controls [38, 39]. Although no mechanism for the trophic effects of oils has been delineated, it is clear that acute, high dose of dietary lipids derived from plant or animal origins can stimulate small intestinal growth.

## 5. Conclusion

In conclusion, the combination of muricid extract with the cytotoxic chemotherapy agent, 5FU, did not confer any additional intestinal toxicity, but may have some immunomodulatory effects towards neutrophils. Muricid extract has potent chemopreventive properties and represents a novel functional food for the treatment of colorectal cancer. However, it is important to completely characterise any potential side-effects that could be associated with this complementary nutraceutical. Interestingly, sunflower oil treatment partially protected rats against the intestinal toxicity associated with 5FU-induced intestinal mucositis and may represent a broader application of oils to protect against intestinal mucositis.
